# Feeding toxicity and impact of imidacloprid formulation and mixtures with six representative pesticides at residue concentrations on honey bee physiology (*Apis mellifera*)

**DOI:** 10.1371/journal.pone.0178421

**Published:** 2017-06-07

**Authors:** Yu Cheng Zhu, Jianxiu Yao, John Adamczyk, Randall Luttrell

**Affiliations:** 1 USDA-ARS, Stoneville, MS, United States of America; 2 USDA-ARS, Poplarville, MS, United States of America; Institut Sophia Agrobiotech, FRANCE

## Abstract

Imidacloprid is the most widely used insecticide in agriculture. In this study, we used feeding methods to simulate in-hive exposures of formulated imidacloprid (Advise^®^ 2FL) alone and mixtures with six representative pesticides for different classes. Advise, fed at 4.3 mg/L (equal to maximal residue detection of 912 ppb active ingredient [a.i.] in pollen) induced 36% mortality and 56% feeding suppression after 2-week feeding. Treatments with individual Bracket (acephate), Karate (λ-cyhalothrin), Vydate (oxamyl), Domark (tetraconazole), and Roundup (glyphosate) at residue level had a mortality range of 1.3–13.3%, statistically similar to that of control (*P*>0.05). The additive/synergistic toxicity was not detected from binary mixtures of Advise with different classes of pesticides at residue levels. The feeding of the mixture of all seven pesticides increased mortality to 53%, significantly higher than Advise only but still without synergism. Enzymatic data showed that activities of invertase, glutathione S-transferase, and acetylcholinesterase activities in imidacloprid-treated survivors were mostly similar to those found in control. Esterase activity mostly increased, but was significantly suppressed by Bracket (acephate). The immunity-related phenoloxidase activity in imidacloprid-treated survivors tended to be lower, but most treatments were statistically similar to the control. Increase of cytochrome P450 activity was correlated with Advise concentrations and reached significant difference at 56 mg/L (12 ppm a.i.). Our data demonstrated that residue levels of seven pesticide in pollens/hive may not adversely affect honey bees, but long term exclusive ingestion of the maximal residue levels of imidacloprid (912 ppb) and sulfoxaflor (3 ppm a.i.) may induce substantial bee mortality. Rotating with other insecticides is a necessary and practical way to reduce the residue level of any given pesticide.

## Introduction

Honey bee (*Apis mellifera* Linnaeus) is an economically important insect. In United States, more than 2.7 million colonies were maintained and managed by American beekeepers in 2014, producing more than 178 million pounds of honey valued at more than $385 million [1]. More importantly, honey bees, through natural and commercialized pollination service, enhanced crop value by approximately $12 billion annually in the United States [2–3]. Unfortunately, honey bees are not immune to biological and physical threats, and they are currently under tremendous pressures from natural and human interferences, including pests, parasites, pathogens, agrochemicals, and loss of natural forage [4–5]. Honey bees are often adversely impacted (although unintentionally) by farming practices, such as the loss of favorable natural habitats, and direct poisoning from pesticide treatments (especially seed coating and foliar sprays) [6].

The widespread use of transgenic plants in last decade has reduced the use of some insecticides, but it also caused a pest status shift from chewing insects to piercing/sucking insects on row crops. Examples include the polyphagous tarnished plant bug (*Lygus lineolaris*) in cotton and stink bugs (*Acrosternum hilare*, *Nezara viridula*, and *Euschitus servus*) in cotton and soybean [7–8]. This pest status shift, coupled with the development of insecticide resistance in target insects [9–10], has resulted in increased use of insecticide-treated seeds and foliar insecticides to control them in row crops. Currently, a variety of insecticides are available for pest control, including pyrethroids, organophosphates, carbamates, and neonicotinoids. More than forty insecticides are currently recommended by extension specialists in the United States for the chemical control of row crop insects [11–13].

New concerns arose regarding honey bee toxicity from airborne insecticide dust during planting [14] and pesticide residues systemically transferred from seeds (treatment) to pollens and nectar [15] as one of the major factors associated with honey bee declining [5]. Residues of more than 150 pesticides were detected at various levels in wax, pollen, bee, or honey [16–17]. The possible relationships between honey bee colony losses and sublethal effects of pesticide residues have received considerable attention, and published data indicated that pesticide residues may pose adverse impacts on honey bee populations [18–19] or very low to no risk [20–21].

Imidacloprid and other neonicotinoids are agonists of nicotinic acetylcholine receptor (nAChR). They act on the central nervous system by interfering with transmission of stimuli by competing with the natural neurotransmitter acetylcholine. Irreversible and selective binding to insect central nervous system causes paralysis and death by over-stimulation and blockage to nAChR [22]. Imidacloprid was the first synthetic neonicotinoid insecticide commercialized in 1991. It incurs toxicity through contact and oral ingestion, and is effective in controlling sucking insects. Its relatively low mammal toxicity and systemic activity contribute to it being one of the most widely used insecticides in the world [22].

The wide use of a variety of pesticides for crop protection, including formulated mixtures and tank mixing, and the detection of more than 150 pesticide residues in honey, pollen and wax have prompted investigations to study whether and how different pesticides and mixtures impact collectively on honey bees [23–29]. In this study, we focused on one of the imidacloprid commercial products, Advise^®^ 2FL (Advise), to measure its relatively long term sublethal impacts on honey bee workers, and examine bees’ biological and physiological responses at sublethal concentration of 4.3 mg/L (equal to maximum residual levels detected in pollen [16–17]). Mixtures of Advise with four representative insecticide classes, one fungicide, and one herbicide at pollen residual levels were used to reveal potential interactions (additive/synergistic toxicity) with imidacloprid. Several detoxification-, metabolic-, and immune-related enzymatic activities were measured after treatment.

## Methods and materials

### 2.1. Honey bee colonies

Honey bee colonies were originally purchased from bee keepers located in pine forest and pasture area in Perkinston and Magee, Mississippi and maintained in a managed bee yard at the Mississippi Wildlife Management Area near Stoneville, MS. An oil trap (35x45 cm tray filled with vegetable oil) was installed at the bottom of each colony for *Varroa* mite (*Varroa destructor*) and small hive beetle (*Aethina tumida*) monitoring and control. Deep frames with more than 50% coverage of healthy sealed brood were pulled out and transferred to laboratory incubators (33°C±0.5°C; 65%±3 RH; no light).

### 2.2. Pesticides and test concentrations

Formulated imidacloprid Advise^®^ 2FL (Advise) and other pesticides were purchased from local agricultural chemical stores and kept in a refrigerator (6±1°C). Sublethal dose or residue levels were determined mostly according to the maximal detection levels by Johnson et al. [16] and Mullin et al. [17] from pollen, wax, bees, and honey inside bee hive. Other internet source (http://www.fao.org/docrep/009/a0209e/a0209e0d.htm) and EPA documentation (EPA Docket # EPA-HQ-OPP-2010-0889 http://www.beyondpesticides.org/assets/media/documents/documents/sulfoxaflorEPAresponse.pdf) were also referred for the concentrations of glyphosate and sulfoxaflor used in this study. The concentrations used for feeding treatments were: Advise (imidacloprid) at 4.3 mg/L, Bracket (acephate) at 0.168 mg/L, Karate (λ-cyhalothrin) at 7.3 mg/L, Vydate (oxamyl) at 0.179 mg/L, Domark (tetraconazole [a fungicide]) at 0.084 mg/L, Roundup (glyphosate [a herbicide]) at 35 mg/L, Transform (sulfoxaflor) at 6 mg/L, 697-, 49036-, 213-, 57531-, 40119-, 2675-, 235-fold lower than field use concentrations, respectively. Details of pesticide name, manufacturer, percentage of active ingredient (a.i.), residue level in pollen, feeding treatment concentration, field use (spray) concentration of formulation, and mode of action were listed in Table 1 [30–33].

**Table 1:** Pesticide name, manufacturer, percentage of active ingredient (a.i.), residue level in pollen, feeding treatment concentration, field use (spray) concentration of formulation, and mode of action.

Common name	Commercial name	Manufacturers	Active ingredient a.i.%	Residue (ppb a.i.) in pollen	Feeding concentration (formulation mg/L)	Field use concentration mg/L [30]	Mode of action
Imidacloprid	Advise 2FL	Winfield Solutions LLC	0.214	912	4.3	2996	Nicotinic acetylcholine receptor (nAChR) competitive modulators [31]
Acephate	Bracket97	Winfield Solutions LLC	0.97	163	0.168	8238	Acetylcholinesterase (AChE) inhibitors [31]
λ-cyhalothrin	Karate Z 2.08 CS	Syngenta	0.228	1670	7.3	1558	Sodium channel modulators [31]
Oxamyl	Vydate 3.77 CLV	DuPont	0.42	75	0.179	10298	Acetylcholinesterase (AChE) inhibitors [31]
Tetraconazole	Domark 230 ME	Valent	0.205	17	0.084	3370	Inhibit ergosterol biosynthesis enzyme C14-demethylase [32]
Glyphosate	Roundup PowerMAX	Monsanto	0.487	17000	35	93614	Glyphosate inhibits 5-enolpyruvylshikimic-3-phosphate synthase (EPSPS), causing a reduction of the biosynthesis of aromatic amino acids [33]
Sulfoxaflor	Transform 5G	Dow AgroSciences	0.5	3000	6	1408	Nicotinic acetylcholine receptor (nAChR) competitive modulators[31]

### 2.3. Bioassay methods

Feeding treatment method was used to simulate in-hive feeding exposure to mixtures of pesticide residues. Advise (imidacloprid) alone and in binary mixtures of Advise with six representative pesticides were incorporated into sugar solution to assess a sublethal effect and potential additive/synergistic toxicity of pesticide mixtures. In this experiment, 30 newly emerged workers were transferred into a cage (made with a 500-ml round wide-mouth polypropylene jar [DxH: 9.3x10 cm]). An 8.9 cm diameter (d) hole was cut in the lid and covered with 3×3 mm-mesh metal screen. Caged bees were provided with 50% sugar solution and water and maintained in incubators (33°C±0.5°C) for three days before being used for testing. Pesticide solution was incorporated into 20 mL 50% sugar solution to make final concentration equal to the concentrations described above (section 2.2 and Table 1). Five replicate cages were used for each treatment. Mortality was recorded at day 7 and day 14. The pesticide-containing sugar solution was replaced with fresh preparations at day 8. The remaining volume of sugar solution was measured at day 7 and day 14, each after 7 days long feeding on sugar solution treated with individual, binary mixtures of Advise with six other pesticides, and a mixture of all seven pesticides. Sugar consumption per bee was calculated by dividing the consumed volume by average number of surviving bees between day 1 and day 7 for the first week and between day 8 and day 14 for the 2^nd^ week. Three surviving bees were collected at day 14 from each cage and were used for enzyme activity assays (below). At the end of synergism feeding test, e.g. after two weeks of feeding on Advise and mixtures with other six pesticides, fresh body weight was measured by using an analytical balance. A group of six surviving workers from each replicate were weighed to calculate average weight per bee.

### 2.4. Enzyme activity assays

#### 2.4.1. Chemicals

The following chemicals were purchased from Sigma-Aldrich (Sigma-Aldrich, St. Louis, MO): protease inhibitor cocktail tablets, α-naphthyl acetate, fast blue B salt, 1-chloro-2,4-dinitrobenzene (CDNB), L-glutathione reduced (GSH), 4-nitrophenyl-α-D-glucopyranoside (pNPG), dopamine hydrochloride, acetylthiocholine iodide (ATC), 5,5`-dithio-bis(2-nitrobenzoic acid) (DTNB), ρ-hydroxybenzhydrazide (PAHBAH), umbelliferone (7-hydroxycoumarin), 7-ethoxycoumarin (7-EC), oxidized glutathione (GSSG), glutathione reductase, β-nicotinamide adenine dinucleotide phosphate (reduced β-NADPH).

#### 2.4.2. Protein preparation

After a week-long feeding period on pesticide-containing sugar solution, midguts (for P450 oxidase) or heads plus thoraxes (for other enzymes) of three surviving workers per replicate (three replicates per treatment) from the synergism (mixture) assay were ground in phosphate buffer pH 7.2 with protease inhibitor and 0.3% Triton X-100. The homogenate was centrifuged at 4°C, 20,800 × g, for 15 min and the supernatant were collected for the following enzyme activity assays described below. Total protein concentration of each enzyme extraction sample was measured by using a Bradford protein assay kit [34] (ThermoScientific. Waltham, MA).

#### 2.4.3. Esterase activity assay

Esterase activity against α-naphthyl acetate was measured using the assay method of Zhu and Gao [35]. Briefly, the homogenate was diluted by 5 fold with 0.1 M phosphate buffer pH 7.5. The reaction solution consisted of 15 μl diluted enzyme and 135 μl 0.3 M α-naphthyl acetate. The reaction solution was incubated at 37°C for 30 min and the reaction was stopped by adding 50 μl fast Blue-SDS. Absorbance values were recorded at 600 nm using a Synergy HTX plate reader (Bio-Tek Instruments Inc. Winooski, VT). The esterase activity was calculated based on the standard linear relationship established using α-naphthol.

#### 2.4.4. Glutathione S-transferase (GST) activity assay

GST activities were determined using the protocols of Yu [36] with some modifications. The reaction solutions (200 μl) contained 10 mM GSH, 2 mM CDNB, and 10 μl enzyme extraction. The optical density (OD) was continuously measured at 340 nm every 15 sec for a total of 10 min on a Synergy HTX plate reader. Specific activity rates were converted to nmol of conjugation min^-1^mg^-1^ protein using experimentally derived extinction coefficients of 5.3 mM^-1^cm^-1^ [37].

#### 2.4.5. Invertase activity assay

Invertase activity was determined using sucrose as substrate according to Lever [38] with some modification [39]. The reaction mixture of 100 μl enzyme extract and 900 μl 1.0% sucrose in 0.1 M pH 4.5 acetate buffer were incubated at 55°C for 20 min. The reaction was stopped by mixing 50 μl of the reaction mixture with 1.45 ml 1% PAHBAH in 0.5 M sodium hydroxide solution and heating the mixture at 95°C for 5 min. The absorbance was measured at 410 nm. Activity of invertase was determined by hydrolyzing 1.0 μmole of sucrose to glucose and fructose min^-1^mg^-1^ protein at 55°C, pH 4.5.

#### 2.4.6. Phenoloxidase activity assay

The reaction solution contained 20 μl of enzyme solution (with hindguts, wings, and legs excluded) and 2 mM dopamine hydrochloride in sodium phosphate buffer pH 6.5. Phenoloxidase activity was measured at 490 nm for 30 min with 30 sec reading interval [40]. Activity of phenoloxidase was defined as the amount of enzyme which causes a change of OD 490 min^-1^mg^-1^ protein in the reaction (units/min/mg protein).

#### 2.4.7. Acetylcholinesterase (AChE) activity assay

AChE activity was measured using acetylthiocholine (ATC) according to the method of Ellman et al. [41] with some modifications. Each reaction mixture included 50 μl enzyme extract, 0.25 mM ATC, and 0.4 mM DTNB in 150 μl of 0.1 M phosphate buffer pH 7.5. The enzyme activity expressed by Vmax mOD/min was determined kinetically at 405 nm using the Synergy HTX plate reader. AChE activities were expressed as nmol ATC hydrolyzed per min per mg protein using the extinction coefficient of 1.36×10^4^ M^−1^ cm^−1^.

#### 2.4.8. Fluorometric determination of cytochrome P450 monooxygenase activity

Three-day-old bees were fed with sugar solution containing (0, 4.3, 22.4, and 55.5 mg/L) Advise for 48 h. The treatment was repeated three times for each concentration. Surviving bees were collected after 48-h feeding. Three midguts, dissected from each replicate, were pooled into 500 μl ice-cold enzyme buffer as one sample and were homogenized and centrifuged at 10,000 × g for 20 min at 4°C. The substrate was collected and was tested for cytochrome P450 monooxygenase activity using 7-EC as a substrate [42] following the methods of Waxman and Chang [43] and Anderson and Zhu [44] with some modifications. In each well, 50 μl of enzyme solution was mixed with 43 μl sodium phosphate buffer (0.1 M, pH 7.2 containing 0.3% Triton and protease inhibitor), 2 μl of 20 mM 7-EC in methanol [45], and 5 μl 20 mM aqueous β-NADPH. The final concentrations of 7 -EC and β-NADPH in each well was 0.4 mM and 1 mM, respectively. The plate was incubated for 30 min at 30°C while shaking at 225 rpm in an Environ Shaker (Lab-Line Instruments Inc. Dubuque, IA). Then, 10 μl GSSG (100 mM in distilled water) and 10 μl glutathione reductase (0.1 unit/μl) were added into each well [46] to oxidize β-NADPH at 15 min at 37°C. Finally, the reaction was stopped with 120 μl of 50% (v/v) acetonitrile in 50 mM TRIZMA-base buffer (pH 10). The fluorescence of 7-hydroxycoumarin was measured with a Synergy HTX plate reader at 460 nm while exciting at 360 nm. The P450 activity (7-EC-O-deethylation) was determined based on 7-hydroxycoumarin standard curve [43] and protein content, and was expressed as nmol of 7-hydroxycoumarin formed per min per mg protein.

### 2.5. Data processing and statistical analysis

SAS (version 9.2) [47] was used for analysis of variance (ANOVA). Proc GLM (general linear model) procedure was applied with option of Fisher’s LSD (least significant difference) method for mean separation at *P* = 0.05.

## Results

### 3.1. Interaction of imidacloprid with fungicide, herbicide, and other insecticides

**Fig 1:**
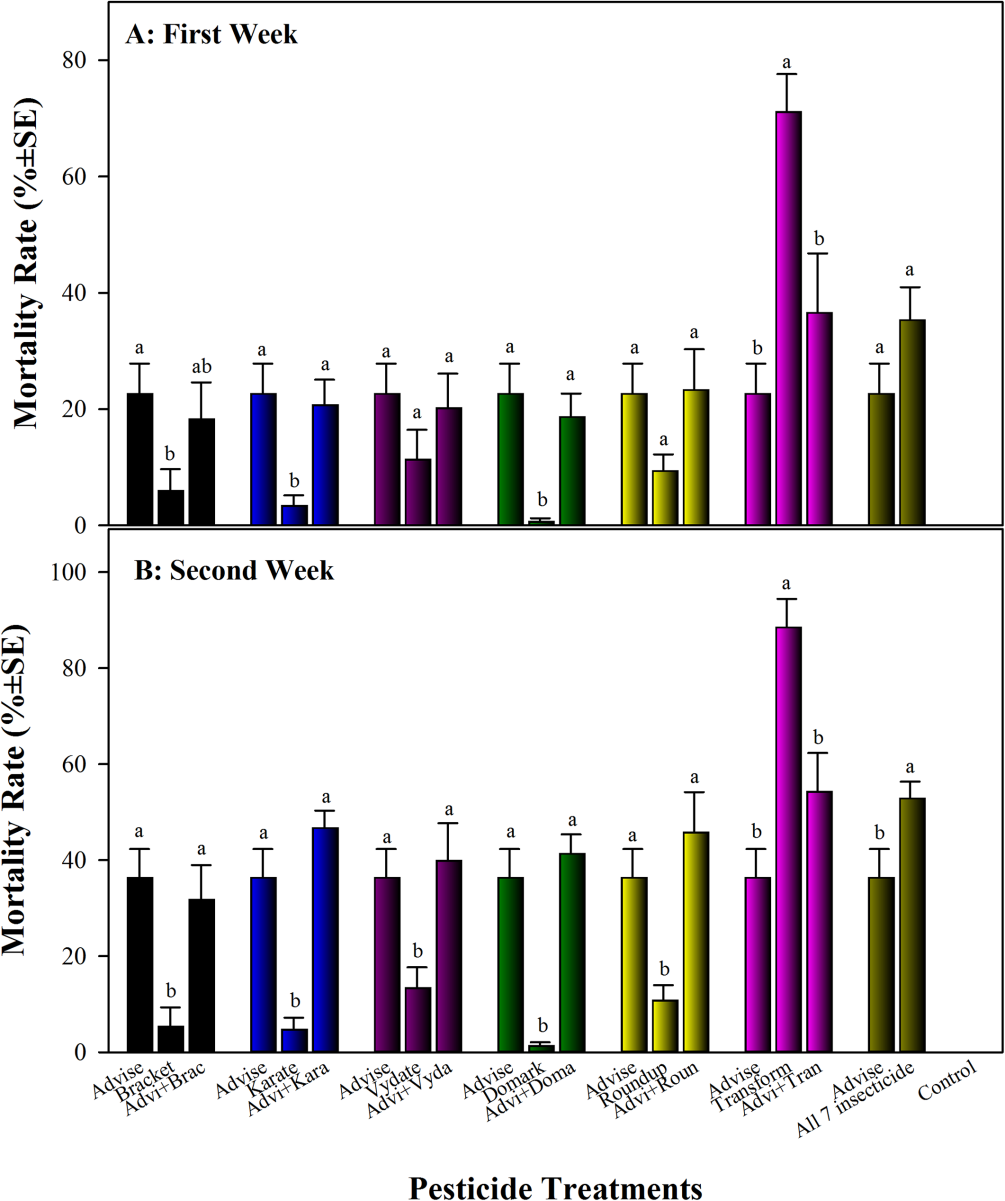
Additive/Synergistic feeding toxicity of Advise (imidacloprid) only and mixtures with six representative pesticides at residue levels (Advise at 4.3 mg/L; Bracket97 [acephate] at 0.168 mg/L; Karate [λ-cyhalothrin] at 7.3 mg/L; Vydate [oxamyl] at 0.179 mg/L; Domark [tetraconazole] at 0.084 mg/L; Roundup [glyphosate] at 35 mg/L; Transform [sulfoxaflor] at 6 mg/L). Within mixture group, the mean bars with same letters at the top of error bars indicate no significant difference, and the mortality data from Advise only was reused in each group for in-group comparison and statistics. A: Mortality after one week of feeding on pesticide-containing sugar solution; B: Mortality after two weeks of feeding on pesticide-containing sugar solution (freshly prepared each week).

After feeding on pesticide-containing sugar solution for one and two weeks, natural mortality in control was zero. Imidacloprid (Advise at 4.3 mg/L) had 22.6% and 36.3% bee mortality, respectively (Fig 1A and 1B), significantly higher than that of water-only control (*P*<0.05). Treatments with Bracket (acephate), Karate (λ-cyhalothrin), Vydate (oxamyl), Domark (tetraconazole), and Roundup (glyphosate) alone had less than 14% mortality (1.3–13.3%), statistically similar to that of control (*P*>0.05). The binary mixtures of Advise (imidacloprid) with these five pesticides produced 32–47% bee mortality, but none of those were significantly higher than Advise only (Fig 1B). After one and two weeks, Transform (sulfoxaflor) alone had 71% and 88% bee mortality, respectively, significantly higher than that of Advise only and the mixtures of Advise with Transform. The mortality test on Advise and Transform was repeated and similar results were obtained. Advise only, transform only, and the mixture of the two insecticides incurred 41%, 79%, and 51% bee mortality, respectively. The mixture of Advise together with all other six pesticides generated 53% bee mortality which was numerically greater than most individual pesticide and mixtures with Advise, except Transform (Fig 1).

### 3.2. Influence of imidacloprid on honey bee feeding and body weight

**Fig 2:**
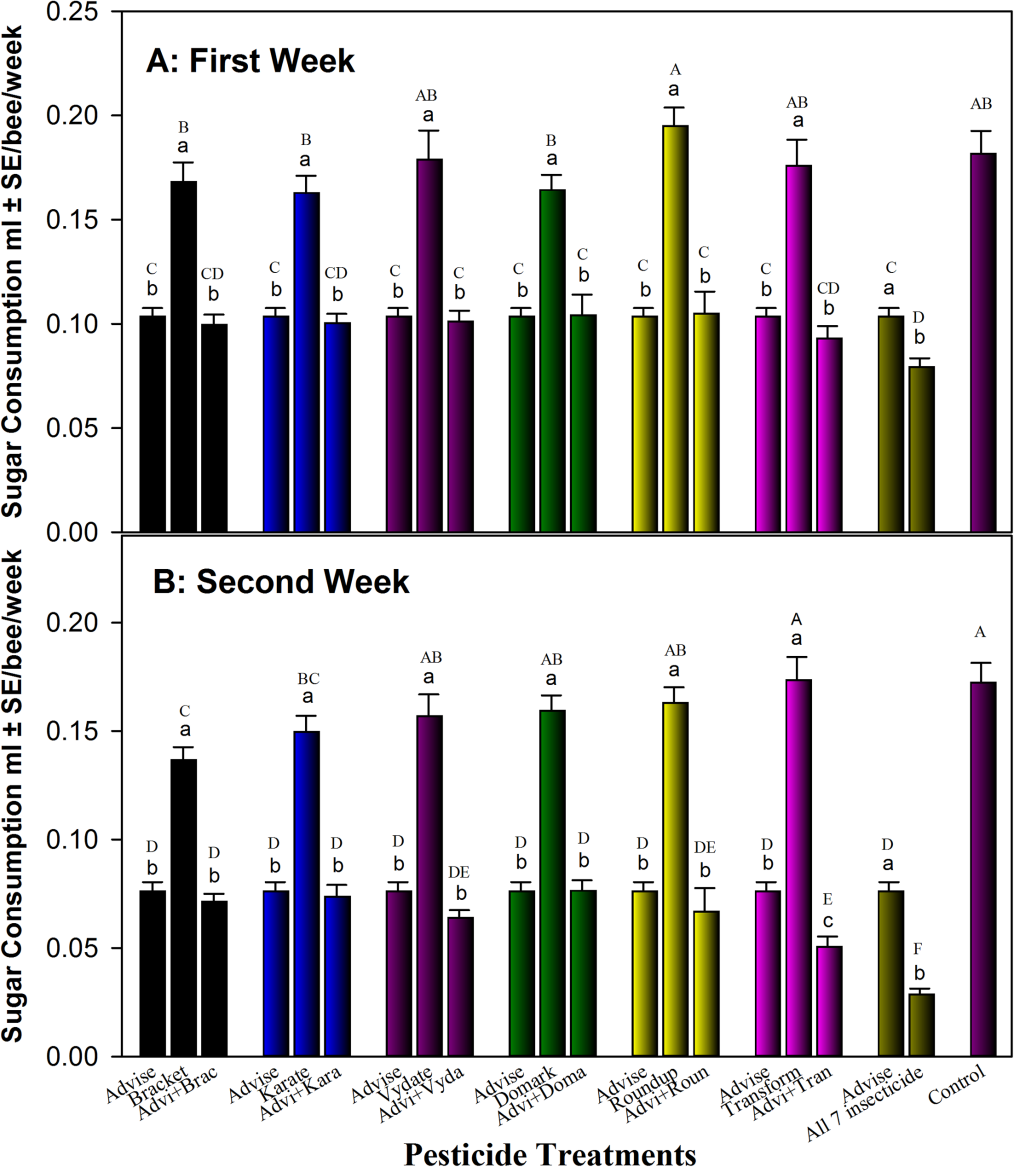
Influence of pesticide treatment on honey bee feeding with Advise (imidacloprid) only and mixtures with six representative pesticides at residue levels (Advise [imidacloprid] at 4.3 mg/L; Bracket97 [acephate] at 0.168 mg/L; Karate [λ-cyhalothrin] at 7.3 mg/L; Vydate [oxamyl] at 0.179 mg/L; Domark [tetraconazole] at 0.084 mg/L; Roundup [glyphosate] at 35 mg/L; Transform [sulfoxaflor] at 6 mg/L). Low case letters were used for mean separation within mixture group, while the up case letters for all treatments. The mortality data from Advise only was reused in each group for each comparison and statistics. Mean bars with same letters at the top of error bars indicate no significant difference. A: Sugar solution consumption after one week; B: Sugar solution consumption after two weeks (solution were freshly prepared each week).

Individual and binary mixtures of Advise (imidacloprid) with the other pesticides were assessed for their impact on honey bee feeding. Results (Fig 2) showed that bees fed Vydate (oxamyl)-only (0.1789/0.1569 mL/bee/week), Roundup (glyphosate)-only (0.1949/0.1629 mL/bee/week), and Transform (sulfoxaflor)-only (0.1758/0.1734 mL/bee/week) consumed similar volume of pesticide-containing sugar solution as control (0.1817/0.1722 mL/bee/week) during the first week and second weeks. Treatments with Bracket (acephate)-only (0.1682/0.1367 mL/bee/week), Karate (λ-cyhalothrin)-only (0.1629/0.0.1495 mL/bee/week), and Domark (tetraconazole)-only (0.1643/0.1593 mL/bee/week) had certain reduction of sugar solution feeding. However, bees fed 43% and 56% less Advise-containing solution at 4.3 mg/L than the bees fed with sugar-solution-only control in week 1 and week 2, respectively. Moreover, all Advise-containing mixtures reduced sugar feeding to the levels similar or significantly lower than Advise only, especially the mixture of all seven pesticides (0.0792/0.0285 mL/bee/week) (Fig 2A and 2B).

**Fig 3:**
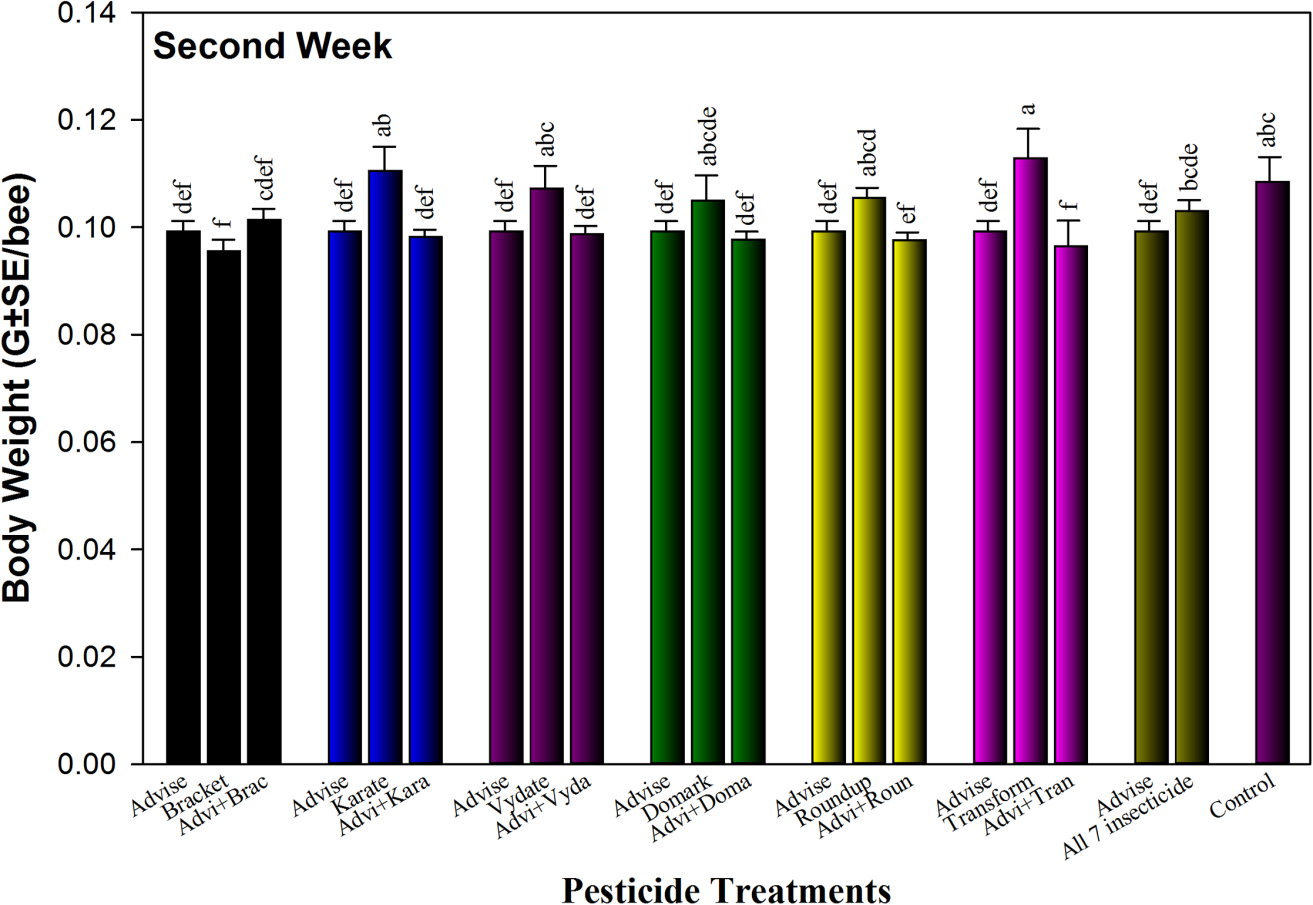
Influence of pesticide treatments on honey bee body weight after two weeks of feeding on Advise (imidacloprid) only and mixtures with six representative pesticides at residue levels (Advise at 4.3 mg/L; Bracket97 [acephate] at 0.168 mg/L; Karate [λ-cyhalothrin] at 7.3 mg/L; Vydate [oxamyl] at 0.179 mg/L; Domark [tetraconazole] at 0.084 mg/L; Roundup [glyphosate] at 35 mg/L; Transform [sulfoxaflor] at 6 mg/L. Mean bars with same letters at the top of error bars indicate no significant difference. The corrected mortality from Advise only was reused in each group for each comparison and statistics.

Body weight data (Fig 3) showed the influences of Advise (imidacloprid) and binary mixtures with six other pesticides at sublethal residue levels. Bees treated with Advise-only had significantly lower body weight than control after 2 weeks. Workers fed on Bracket- and Advise+Transform-treated sugar solution had lowest body weight. Results also indicated that any Advise-containing sugar solutions induced body weight reduction, except for bees exposed to the mixture with Bracket and the mixture of Advise with all six pesticides.

### 3.3. Pesticide impact on honey bee detoxification systems

#### 3.3.1. Influence of imidacloprid and mixtures on esterase (EST) activity

**Fig 4:**
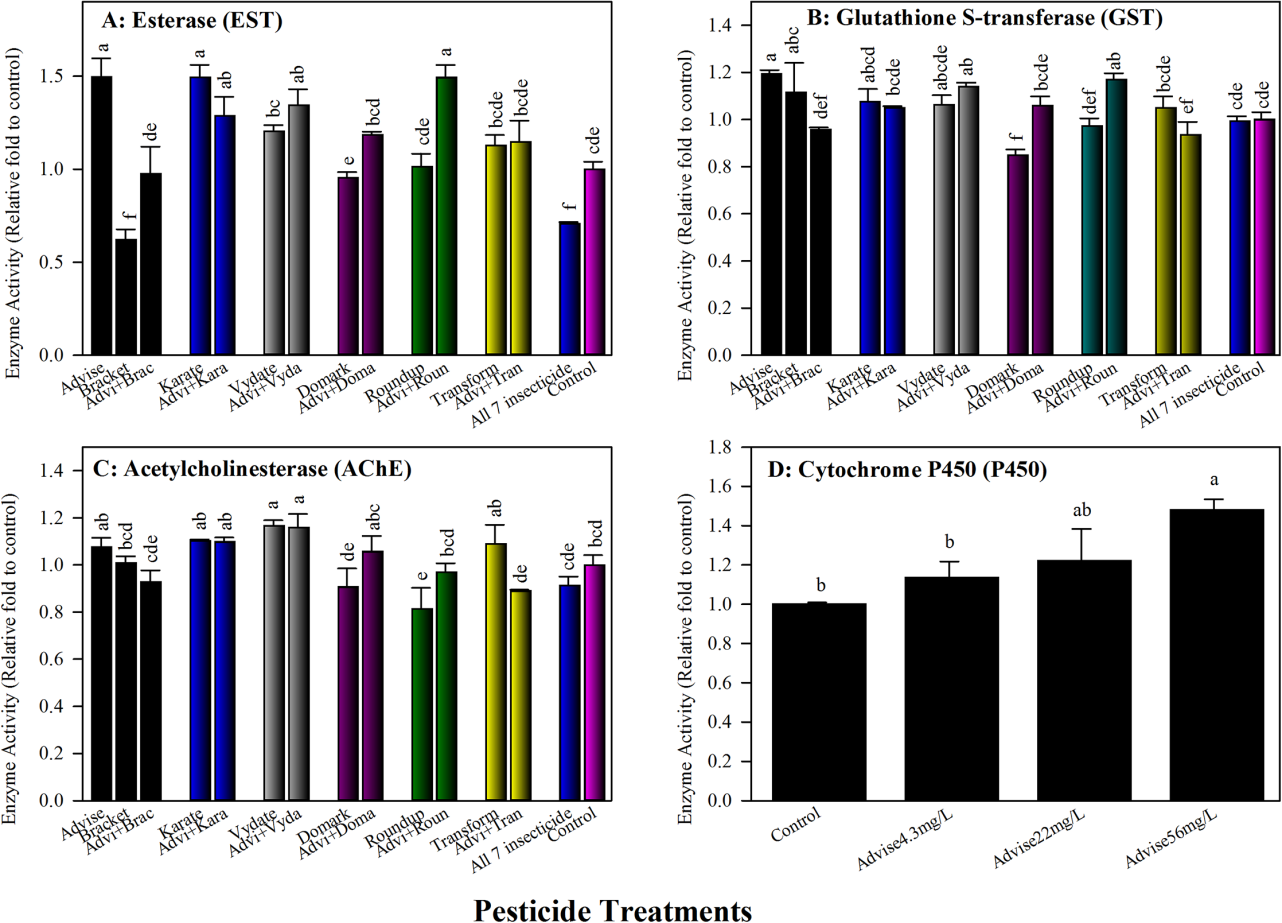
Impact of pesticide treatments on insecticide-targeting enzyme and defense-related enzymes in bees fed two weeks with Advise (imidacloprid) only and mixtures with six representative pesticides at residue levels (Advise at 4.3 mg/L; Bracket97 [acephate] at 0.168 mg/L; Karate [λ-cyhalothrin] at 7.3 mg/L; Vydate [oxamyl] at 0.179 mg/L; Domark [tetraconazole] at 0.084 mg/L; Roundup [glyphosate] at 35 mg/L; Transform [sulfoxaflor] at 6 mg/L). Mean bars with same letters at the top of error bars indicate no significant difference. A: Esterase (EST); B: Glutathione S-transferase (GST); C: Acetylcholinesterase (AChE), and D: Cytochrome P450 oxidase (P450).

Enzyme activity data were expressed in relative ratios of treatment to control for comparison across the data obtained from different enzyme preparations, each of those containing a control. Advise (imidacloprid) alone at 4.3 mg/L significantly increased EST activity by 50%, while Bracket (acephate) at 0.168 mg/L significantly suppressed EST activity by nearly 40% in honey bee workers (Fig 4A). Bees treated with the mixture of Advise and Bracket had intermediate EST activity, which was similar to that of control. The other pesticides alone and binary mixtures with Advise produced EST activities either significantly higher or similar to the activity of control. Mixture of all seven pesticides, similar to Bracket only, significantly suppressed esterase activity.

#### 3.3.2. Influence of imidacloprid and mixtures on glutathione S-transferase (GST) activity

Domark (a fungicide) was the only pesticide tested that significantly suppressed GST activity in honey bee workers (Fig 4B). All other treatments alone and in binary mixtures with Advise (imidacloprid) generated significantly higher or similar GST activities to that of control. The mixture of all seven pesticides also had similar GST activity as control (Fig 4B).

#### 3.3.3. Influence of imidacloprid and mixtures on acetylcholinesterase (AChE) activity

Vydate (oxamyl), a carbamate insecticide, was the only pesticide inducing significantly higher AChE activity individually and collectively with Advise (Fig 4C). While Roundup (herbicide) alone was the only treatment that significantly suppressed AChE activity in bees. All other treatments, Advise only and binary mixtures with the other pesticides tested produced similar AChE activity to that of control. Similarly, the mixture of all seven pesticides also showed no effect on AChE activity.

#### 3.3.4. Influence of imidacloprid on cytochrome P450 oxidase (P450) activity

Three concentrations of Advise (imidacloprid) were examined for their impact on P450 activity in honey bee workers, including the 4.3 mg/L, the maximal residue levels detected from pollens. Results (Fig 4D) showed that the P450 activity increased as treatment concentrations increased. The increase of P450 activity reached statistically significant level as Advise concentration was raised to 56 mg/L.

### 3.4. Pesticide impact on honey bee metabolic and immune systems

#### 3.4.1. Influence of imidacloprid and pesticide mixtures on invertase (INV) activity

**Fig 5:**
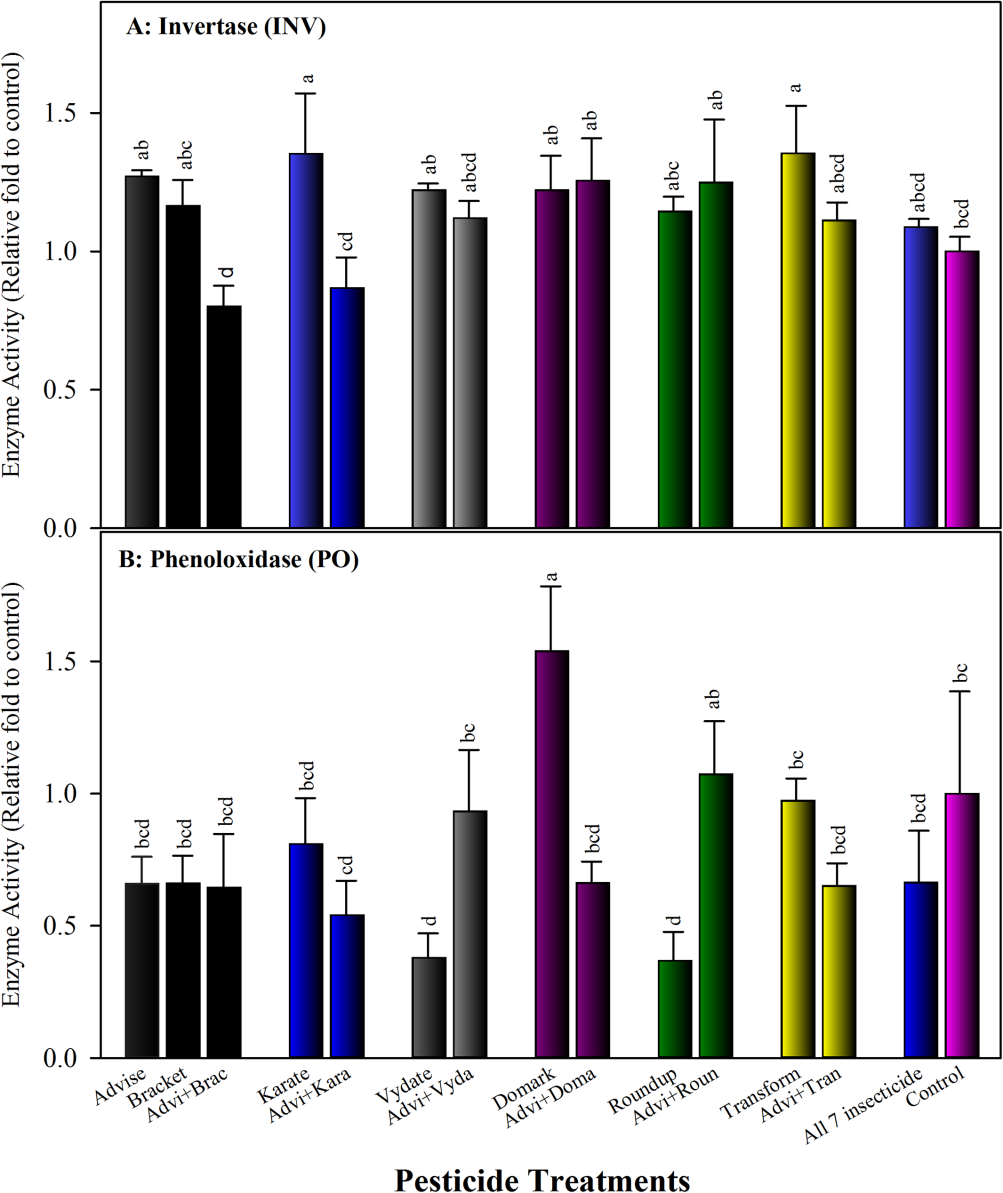
Impact of pesticide treatments on metabolic- and immune-related enzymes in bees fed two weeks with Advise (imidacloprid) only and mixtures with six representative pesticides at residue levels (Advise at 4.3 mg/L; Bracket97 [acephate] at 0.168 mg/L; Karate [λ-cyhalothrin] at 7.3 mg/L; Vydate [oxamyl] at 0.179 mg/L; Domark [tetraconazole] at 0.084 mg/L; Roundup [glyphosate] at 35 mg/L; Transform [sulfoxaflor] at 6 mg/L). Mean bars with same letters at the top of error bars indicate no significant difference. A: Invertase (INV); and B: Phenoloxidase (PO).

Both Karate (λ-cyhalothrin) at 7.3 mg/L alone and Transform (Sulfoxaflor) at 6 mg/L alone induced significantly higher INV activity. All other treatments of individual pesticides and mixtures did not increase or reduce invertase activity significantly (Fig 5A). It is notable that the binary mixture of Advise (imidacloprid) and Bracket produced significantly reduced INV activity. Similarly, the mixture of Advise with Karate significantly lowed INV activity. The mixture of all seven pesticides induced slightly higher INV activity than control.

#### 3.4.2. Influence of imidacloprid and pesticide mixtures on phenoloxidase (PO) activity

In general, PO activities were numerally lower than that of control, but most treatments produced statistically similar PO activity as that of control. Domark (fungicide tetraconazole) induced significantly higher PO activity, while Vydate (oxamyl) alone and Roundup (herbicide glyphosate) alone showed significantly lower PO activities than the control (Fig 5B). Advise (imidacloprid) only reduced PO activity by approximately 35%, and most binary mixtures with other pesticides produced similar PO activities as Advise only, including the mixture of all seven pesticides (Fig 5B).

## Discussion

Currently sublethal pesticide residues in pollens is a major concern for the honey bee colony decline, especially neonicotinoids that are widely used for seed treatment [48] and foliar spray. To better assess insecticide toxicity risk in honey bees, we simulated in-hive exposures and treated bees by feeding methods in this study with an attempt to understand sublethal toxicities of imidacloprid and binary mixtures with six other pesticides representing different pesticide classes. We also used commercial imidacloprid formulation Advise (normally used by farmers) instead of technical grade of imidacloprid to include potential additive and/or synergistic toxicity to bees from formulating reagents [49–50]. Data from this study provided some valuable information regarding **(1)** whether long term exposure to pesticide residues is safe to bees and **(2)** whether pesticide residues from multiple pesticide classes synergized the toxicity to bees. In addition, **(3)** the multiple enzyme activity data shed new light on the understanding of toxicological impact of pesticides on honey bee physiology.

First, we continuously treated honey bee workers for two weeks by feeding sugar solutions spiked with residue concentrations of seven different pesticides (classes) individually or in combinations with Advise (imidacloprid) in attempt to answer an important question regarding whether these pesticide residues are safe (not lethal) to honey bees. Many studies have been done previously but clear conclusion is usually hard to draw. Reports indicated that residue levels of imidacloprid from seed-dressings pose only a negligible risk to honey bees [51–53]. But other reports [54–55] suggested that imidacloprid could impair learning, reduce expected performance and winter survival [56–57], delay returning or even completely disappear from feeders treated with imidacloprid [58–59]. Most of these researches did not include direct measurement of honey bee mortality. Our data clearly indicated that the residues of Bracket (acephate), Karate (λ-cyhalothrin), Vydate (oxamyl), Domark (tetraconazole), and Roundup (glyphosate) are not lethal. Although these pesticides induced 1.3–13.3% mortality after continuous feeding treatment for two weeks, the effect was not significantly different from that of control (*P*>0.05). However, week-long exclusive feeding on imidacloprid-contaminated sugar (912 μg a.i./L) may cause 36% bee mortality. But this extreme concentration (912 ppb) was not detected all times and average residue level was only 3.1 ppb in pollens [17]. In addition, Transform at 6 mg/L (equal to 3 ppm a.i. of sulfoxaflor) is not safe to bees. Up to 88.5% bees died after 2-week feeding on Transform-containing sugar solution. Similarly, the residue of 3 ppm sulfoxaflor was the maximal detection level from pollens and most residue analyses (samples) showed a range of 0.1 to 1 ppm (EPA Docket # EPA-HQ-OPP-2010-0889 http://www.beyondpesticides.org/assets/media/documents/documents/sulfoxaflorEPAresponse.pdf). Further investigations are needed to determine what residue levels of sulfoxaflor and other commonly used pesticides are safe to honey bees.

We also made it clear in this study that Advise (imidacloprid) alone and binary mixtures with six representative pesticides all at residue level concentrations did not induce obvious additive/synergistic toxicity against honey bee workers. In contrary and interestingly, the mixture of Advise+Transform incurred significantly lower mortality than Transform alone. The antagonism might be caused by inhibitory effect on feeding from Advise. Previously, only limited studies have been done on synergistic toxicity of pesticide mixtures against honey bees, and published data showed significantly higher toxicity in mixtures than those in individual chemicals [27–28, 49]. In this study, we did differently with focus on a neonicotinoid insecticide Advise (imidacloprid, nicotinic acetylcholine receptor [nAChR] competitive modulators [31]) the most widely used insecticide and one of the most concerning neonicotinoid insecticides potentially related to honey bee declining. Six pesticides for mixtures represent six different pesticide classes, including a fungicide Domark (tetraconazole) and an herbicide Roundup (glyphosate). The binary mixtures of two different pesticide classes may be more likely to induce synergistic toxicity because they have different mode of action and different target sites [31,60]. However, mortality data from this study indicated that imidacloprid alone and mixtures with five representative insecticides generated similar mortality, while these insecticides alone produced very low or no mortality except sulfoxaflor (Transform). Only a minor additive toxicity (~10% increase, but not statistically significant) was observed in the mixtures of imidacloprid with all other five pesticides except for the mixture with Transform. All of these data indicated that none or very minor additive toxicity was found in the mixtures of Advise with other pesticides at concentrations similar to the residue levels detected in honey bee hives [16–17]. However, we do not exclude that synergism may occur in other situations, such as the mixtures containing higher chemical concentrations or different proportions of individual chemicals in mixture.

Besides the mortality data from additive/synergistic toxicity test, we also found that honey bees ingested significantly (approximately 56%) less sugar solutions, any of that contained residue level of imidacloprid (4.3 mg/L of Advise). Possibly as the consequence of feeding inhibition [61–62] from Advise, honey bee body weight was substantially reduced in bees fed with Advise-containing sugar solutions.

Finally, we examined multiple enzyme activities including 3 pesticide detoxification enzymes (EST, GST, and P450), one insecticide target enzyme (AChE), one honey-making enzyme (INV), and one immunity enzyme (PO) in honey bee survivors after exposures to Advise (imidacloprid) and mixtures. Esterases (ESTs) in insects are frequently implicated in the detoxification or resistance to organophosphates, carbamates, and pyrethroids mainly through gene amplification and upregulation [63]. Glutathione S-transferases (GSTs) catalyze the secondary metabolism of a vast array of compounds oxidized by the cytochrome P450 family [64]. The catalysis reactions transform a wide range of endogenous and xenobiotic compounds, including herbicides and insecticides [65]. ESTs and GSTs have not been well studied in honey bees. Limited data indicated that ESTs and GSTs appeared to be less important in honey bee detoxification system [29]. In this study, we found that most pesticide treatments increased EST and GST activities. The enzymatic data were obtained from survivors and the increase of esterase and GST activity may contribute to the detoxification and surviving. Cytochrome P450 oxidase (P450) is another important detoxification enzyme. How P450 genes are associated with imidacloprid detoxification has not been well established in honey bees, although P450 inhibitors effectively increased toxicity of cyano-group neonicotinoids to bees but not to imidacloprid (nitro-group) [29]. It is possible that imidacloprid metabolites (5- hydroxyimidacloprid and olefin) have high affinity for the honey bee nAChR to induce bee mortality [66–67], and P450s may be still responsible for the production of these metabolites, although the metabolic process took longer than that for cyano-group neonicotinoids [29, 68]. Nevertheless, P450s have not been excluded in imidacloprid detoxification. The demonstration of increased P450 activity associated with increasing of Advise (imidacloprid) concentrations in this study indicated that further studies to identify specific P450 genes responsible for particular pesticide detoxification and to develop specific biomarkers for assessing toxicity risk of pesticides in honey bees are needed.

Acetylcholinesterase (AChE) inactivates the neurotransmitter acetylcholine in the synapses of the insect central nervous system [68–69]. In this study, we detected relatively higher AChE activity in Advise (imidacloprid)- and Transform-treated bees and lower AChE activity in Roundup-treated bees, similar to those reported by Boily et al. [70]. Bracket (organophosphate) and Vydate (carbamate) did not inhibit AChE activity in this study. These results differed from Casida and Durkin’s conclusion of inhibitory effects on AChE activity by both organophosphate (OP) and methylcarbamate (MC) insecticides [69]. But in some cases, poor AChE-inhibition and reduced toxicity of OP and MC insecticides against honey bees were observed by Camp et al. [71]. Johnson [68] further suggested that the poor inhibitory activity against the honey bee AChE was possibly associated with development of the tolerance to particular MCs and OPs or by detoxification, rather than bioactivation, through cytochrome P450 oxidases [68,72]. Therefore, lacking significant AChE inhibitions in Bracket- and Vydate-treated bees in this study might be caused by the P450 detoxification. In addition, feeding bees with residue concentration Bracket (0.168 mg/L) and Vydate (0.179 mg/L) might be not high enough to exert inhibition on AChE because these concentrations were 49,036- and 57,531-fold lower than recommended field use concentration for Bracket and Vydate, respectively. Pesticide degradation may also contribute to the difference, but the degradation seemed less important, because the same enzyme preparation from Bracket-treated bees was used for both esterase and AChE activity assays and only esterase was inhibited, suggesting different sensibilities of different enzymes to an inhibitor.

We further examined impact of imidacloprid on invertase, the most important enzyme in honey being responsible for the hydrolysis of nectar sucrose with the formation of fructose and glucose [73]. Detecting no significant inhibition on invertase activity in this study demonstrated that individual and binary mixtures of Advise (imidacloprid) and six pesticides at residue levels had no adverse impact on invertase. We also examined how imidacloprid and mixtures impacted phenoloxidase, which is a key component of the insect immune system [74]. Collectively, phenoloxidase activity in survivors tend to be numerically lower than control after two weeks feeding of pesticide-containing sugar solutions, but most treatments produced statistically similar PO activities as observed in control. Potential link between diseases and/or parasites in bees and neonicotinoids and other pesticides [75–76] and the detection of relatively variable PO activities among treatments necessitate further investigation to understand what factors contributing to the change of PO activities, such as *Varroa* and/or viral infestations.

In summary, pesticide residues in plants and bee hives have been a concern to those interested in honey bees, and existence of multiple pesticide residues in bee hive may incur additive and synergistic toxicity, which is a serious risk to bee and human health. This study focused on biological and physiological impact of sublethal and synergistic toxicity from imidacloprid (the most widely used neonicotinoids) and mixtures with 6 representative pesticides on honey bee workers. Our data indicated that long term exposure to imidacloprid at 912 ppb (the maximal detected residue level from pollen) incurred 36% mortality after two-week long feeding on imidacloprid-containing sugar solution, providing a warning and potential strategy to reduce the residue by rotation with alternative insecticides. The binary mixtures of imidacloprid with six representative pesticides from different classes did not induce any additive/synergistic toxicity, although significant higher mortality than imidacloprid alone was observed in the mixture of all seven pesticides together. Except for a significant suppression of esterase activity by an organophosphate (acephate), three detoxification enzymes (EST, GST, and P450), one insecticide target enzyme (AChE), one honey making enzyme (INV), and one immunity enzyme (PO) were not significantly suppressed by imidacloprid alone or mixtures with six representative pesticides. All of these data indicated that honey bees can tolerant certain levels of pesticide residues in their habitat. Although our data already shed light on many concerning issues, especially the synergistic toxicity from pesticide mixtures and physiological impact, we are continuing investigations on whether and how imidacloprid at high concentrations interacts with other pesticides to synergize toxicity and impact on honey bee physiology. We are also expanding the interactions to examine how *Varroa* mite/viral infestations increase pesticide susceptibility.

## Supporting information

S1 Data SupData_Sublethal7PesticFeeding.pdf. (PDF)
