# Supplementary material for: Feeding toxicity and impact of imidacloprid formulation and mixtures with six representative pesticides at residue concentrations on honey bee physiology (Apis mellifera)
Source: PLoS One. 2017 Jun 7;12(6):e0178421. doi: 10.1371/journal.pone.0178421 (PMC5462352; doi:10.1371/journal.pone.0178421)
Supplement: S1 Data — (PDF) [file pone.0178421.s001.pdf]

Bioassay Data

input treat $ rep mix $ wk1mort wk2mort wk1suga wk2suga GperBee @;

cards;

ck	1	mix0	0	0	0.19712	0.15329	0.11228
ck	2	mix0	0	0	0.17139	0.16194	0.09752
ck	3	mix0	0	0	0.14418	0.16011	0.1189
ck	4	mix0	0	0	0.19043	0.20393	0.11627
ck	5	mix0	0	0	0.2054	0.18165	0.0971
AdviB	1	mix1	0.28125	0.375	0.10718	0.06648	0.09982
AdviB	2	mix1	0.23333	0.46667	0.10297	0.07507	0.09368
AdviB	3	mix1	0.3871	0.51613	0.10721	0.06721	0.09863
AdviB	4	mix1	0.1	0.2	0.11194	0.08483	0.10563
AdviB	5	mix1	0.12903	0.25806	0.08851	0.08689	0.09857
Brac	1	mix1	0	-0.03333	0.16334	0.1312	0.09148
Brac	2	mix1	0.06667	0.06667	0.13877	0.12984	0.09703
Brac	3	mix1	0	0	0.16383	0.13954	0.10255
Brac	4	mix1	0.2	0.2	0.19403	0.12479	0.09023
Brac	5	mix1	0.03226	0.03226	0.18106	0.1582	0.09618
AdviBrac	1	mix1	0.23333	0.36667	0.09075	0.06015	0.10917
AdviBrac	2	mix1	0.34483	0.55172	0.09784	0.07572	0.09887
AdviBrac	3	mix1	0.03333	0.2	0.08838	0.06629	0.10212
AdviBrac	4	mix1	0.26667	0.33333	0.11367	0.07816	0.09745
AdviBrac	5	mix1	0.03448	0.13793	0.10739	0.07689	0.09912
AdviK	1	mix2	0.28125	0.375	0.10718	0.06648	0.09982
AdviK	2	mix2	0.23333	0.46667	0.10297	0.07507	0.09368
AdviK	3	mix2	0.3871	0.51613	0.10721	0.06721	0.09863
AdviK	4	mix2	0.1	0.2	0.11194	0.08483	0.10563
AdviK	5	mix2	0.12903	0.25806	0.08851	0.08689	0.09857
Kara	1	mix2	0.03448	0.03448	0.18373	0.14382	0.12772
Kara	2	mix2	0	0	0.14143	0.12443	0.10985
Kara	3	mix2	0.1	0.13333	0.17871	0.1661	0.10522
Kara	4	mix2	0.03333	0.06667	0.16249	0.16335	0.10828
Kara	5	mix2	0	0	0.14791	0.1498	0.1012
AdviKara	1	mix2	0.36667	0.53333	0.09816	0.08623	0.09505
AdviKara	2	mix2	0.2	0.46667	0.10556	0.08022	0.10037
AdviKara	3	mix2	0.16667	0.53333	0.10894	0.0597	0.09535
AdviKara	4	mix2	0.1	0.46667	0.0841	0.06114	0.10163
AdviKara	5	mix2	0.2	0.33333	0.10526	0.08063	0.09872
AdviV	1	mix3	0.28125	0.375	0.10718	0.06648	0.09982
AdviV	2	mix3	0.23333	0.46667	0.10297	0.07507	0.09368
AdviV	3	mix3	0.3871	0.51613	0.10721	0.06721	0.09863
AdviV	4	mix3	0.1	0.2	0.11194	0.08483	0.10563
AdviV	5	mix3	0.12903	0.25806	0.08851	0.08689	0.09857
Vyda	1	mix3	0	0	0.14791	0.14197	0.10813
Vyda	2	mix3	0.26667	0.26667	0.21738	0.16565	0.11997
Vyda	3	mix3	0	0.1	0.15843	0.12615	0.10722
Vyda	4	mix3	0.13333	0.13333	0.20706	0.16884	0.10737
Vyda	5	mix3	0.16667	0.16667	0.16371	0.18168	0.0933
AdviVyda	1	mix3	0.2069	0.51724	0.10153	0.0583	0.09425
AdviVyda	2	mix3	0.29032	0.35484	0.08525	0.06025	0.09537
AdviVyda	3	mix3	0.16667	0.33333	0.11748	0.07569	0.10145
AdviVyda	4	mix3	0.34483	0.62069	0.10324	0.06894	0.10135
AdviVyda	5	mix3	0	0.16667	0.09825	0.05624	0.10093
AdviD	1	mix4	0.28125	0.375	0.10718	0.06648	0.09982
AdviD	2	mix4	0.23333	0.46667	0.10297	0.07507	0.09368
AdviD	3	mix4	0.3871	0.51613	0.10721	0.06721	0.09863
AdviD	4	mix4	0.1	0.2	0.11194	0.08483	0.10563
AdviD	5	mix4	0.12903	0.25806	0.08851	0.08689	0.09857
Doma	1	mix4	0.03125	0.03125	0.15218	0.16324	0.11768
Doma	2	mix4	0	0	0.14926	0.14089	0.10948
Doma	3	mix4	0	0.03333	0.15735	0.15206	0.09287
Doma	4	mix4	0	0	0.18677	0.15574	0.10927
Doma	5	mix4	0	0	0.17571	0.18435	0.0957
AdviDoma	1	mix4	0.2	0.33333	0.10196	0.07408	0.09505
AdviDoma	2	mix4	0.23333	0.46667	0.13261	0.08878	0.09933
AdviDoma	3	mix4	0.26667	0.5	0.07474	0.06004	0.09363
AdviDoma	4	mix4	0.2	0.46667	0.11726	0.08135	0.1022
AdviDoma	5	mix4	0.03333	0.3	0.09415	0.07793	0.09808
AdviR	1	mix5	0.28125	0.375	0.10718	0.06648	0.09982
AdviR	2	mix5	0.23333	0.46667	0.10297	0.07507	0.09368
AdviR	3	mix5	0.3871	0.51613	0.10721	0.06721	0.09863
AdviR	4	mix5	0.1	0.2	0.11194	0.08483	0.10563
AdviR	5	mix5	0.12903	0.25806	0.08851	0.08689	0.09857
Roun	1	mix5	0.16667	0.2	0.21141	0.15754	0.10097
Roun	2	mix5	0	0	0.17525	0.15095	0.10857
Roun	3	mix5	0.06897	0.10345	0.22042	0.18592	0.11082
Roun	4	mix5	0.13333	0.13333	0.18855	0.17304	0.1024
Roun	5	mix5	0.1	0.1	0.17871	0.14706	0.10482
AdviRoun	1	mix5	0.3	0.6	0.09875	0.06132	0.09895

Enzyme Activity Data

input treat $ rep enzyme $ ActiFold @;

cards;

CK	1	PO	0.70486
CK	2	PO	0.52911
CK	3	PO	1.76604
Advi	1	PO	0.64088
Advi	2	PO	0.84445
Advi	3	PO	0.49218
Brac	1	PO	0.52491
Brac	2	PO	0.58966
Brac	3	PO	0.86659
AdviBrac	1	PO	0.32791
AdviBrac	2	PO	1.02076
AdviBrac	3	PO	0.58685
Kara	1	PO	0.64669
Kara	2	PO	1.15712
Kara	3	PO	0.6238
AdviKara	1	PO	0.78196
AdviKara	2	PO	0.33649
AdviKara	3	PO	0.50301
Vyda	1	PO	0.26166
Vyda	2	PO	0.5599
Vyda	3	PO	0.3171
AdviVyda	1	PO	1.05223
AdviVyda	2	PO	1.26275
AdviVyda	3	PO	0.48256
Doma	1	PO	1.40158
Doma	2	PO	2.01615
Doma	3	PO	1.19902
AdviDoma	1	PO	0.82058
AdviDoma	2	PO	0.60599
AdviDoma	3	PO	0.56055
Roun	1	PO	0.57832
Roun	2	PO	0.21345
Roun	3	PO	0.31281
AdviRoun	1	PO	0.78143
AdviRoun	2	PO	1.4587
AdviRoun	3	PO	0.97839
Tran	1	PO	1.03885
Tran	2	PO	1.07439
Tran	3	PO	0.80554
AdviTran	1	PO	0.59876
AdviTran	2	PO	0.81854
AdviTran	3	PO	0.53407
Mix7	1	PO	0.31623
Mix7	2	PO	0.99301
Mix7	3	PO	0.68259
CK	1	GST	1.03189
CK	2	GST	1.03038
CK	3	GST	0.93772
Advi	1	GST	1.17117
Advi	2	GST	1.22349
Advi	3	GST	1.18955
Brac	1	GST	1.04323
Brac	2	GST	1.35932
Brac	3	GST	0.94396
AdviBrac	1	GST	0.95959
AdviBrac	2	GST	0.97209
AdviBrac	3	GST	0.94061
Kara	1	GST	0.9712
Kara	2	GST	1.10225
Kara	3	GST	1.15278
AdviKara	1	GST	1.06443
AdviKara	2	GST	1.04188
AdviKara	3	GST	1.04313
Vyda	1	GST	1.04404
Vyda	2	GST	1.13968
Vyda	3	GST	1.00452
AdviVyda	1	GST	1.11267
AdviVyda	2	GST	1.13804
AdviVyda	3	GST	1.16924
Doma	1	GST	0.82189
Doma	2	GST	0.82595
Doma	3	GST	0.89789
AdviDoma	1	GST	1.13669
AdviDoma	2	GST	1.03292
AdviDoma	3	GST	1.00778
Roun	1	GST	1.01966

AdviRoun	2	mix5	0.4	0.66667	0.13833	0.10574	0.10047
AdviRoun	3	mix5	0.06452	0.35484	0.07206	0.03951	0.09698
AdviRoun	4	mix5	0.33333	0.46667	0.10656	0.05806	0.09938
AdviRoun	5	mix5	0.06667	0.2	0.10889	0.06906	0.09208
AdviT	1	mix6	0.28125	0.375	0.10718	0.06648	0.09982
AdviT	2	mix6	0.23333	0.46667	0.10297	0.07507	0.09368
AdviT	3	mix6	0.3871	0.51613	0.10721	0.06721	0.09863
AdviT	4	mix6	0.1	0.2	0.11194	0.08483	0.10563
AdviT	5	mix6	0.12903	0.25806	0.08851	0.08689	0.09857
Tran	1	mix6	0.86207	1	0.22279	0.17876	0.10195
Tran	2	mix6	0.73333	0.93333	0.17217	0.20405	0.12022
Tran	3	mix6	0.48276	0.65517	0.1671	0.14418	0.11618
Tran	4	mix6	0.8	0.9	0.17094	0.18526	0.11278
Tran	5	mix6	0.67742	0.93548	0.14614	0.15482	0.11278
AdviTran	1	mix6	0.35484	0.54839	0.114	0.05043	0.10417
AdviTran	2	mix6	0.75862	0.82759	0.07782	0.06619	0.0884
AdviTran	3	mix6	0.21429	0.53571	0.08941	0.03779	0.10987
AdviTran	4	mix6	0.2	0.33333	0.08967	0.04445	0.08437
AdviTran	5	mix6	0.3	0.46667	0.09431	0.05385	0.09572
Advi7	1	mix7	0.28125	0.375	0.10718	0.06648	0.09982
Advi7	2	mix7	0.23333	0.46667	0.10297	0.07507	0.09368
Advi7	3	mix7	0.3871	0.51613	0.10721	0.06721	0.09863
Advi7	4	mix7	0.1	0.2	0.11194	0.08483	0.10563
Advi7	5	mix7	0.12903	0.25806	0.08851	0.08689	0.09857
7ChemMix	1	mix7	0.46667	0.5	0.07358	0.02813	0.09785
7ChemMix	2	mix7	0.36667	0.56667	0.0694	0.02893	0.10635
7ChemMix	3	mix7	0.2069	0.44828	0.07661	0.03277	0.09837
7ChemMix	4	mix7	0.48387	0.64516	0.08235	0.01831	0.1062
7ChemMix	5	mix7	0.24138	0.48276	0.09419	0.03449	0.10648

```
run;
data a1; set a;
```

Roun	2	GST	0.91201
Roun	3	GST	0.98765
AdviRoun	1	GST	1.21701
AdviRoun	2	GST	1.12565
AdviRoun	3	GST	1.16581
Tran	1	GST	1.02776
Tran	2	GST	1.1425
Tran	3	GST	0.97895
AdviTran	1	GST	0.86996
AdviTran	2	GST	0.8881
AdviTran	3	GST	1.04467
Mix7	1	GST	0.95732
Mix7	2	GST	0.99769
Mix7	3	GST	1.02608
CK	1	Est	0.92075
CK	2	Est	1.03262
CK	3	Est	1.04664
Advi	1	Est	1.42912
Advi	2	Est	1.36748
Advi	3	Est	1.69216
Brac	1	Est	0.6202
Brac	2	Est	0.71757
Brac	3	Est	0.52451
AdviBrac	1	Est	0.96362
AdviBrac	2	Est	1.23271
AdviBrac	3	Est	0.73245
Kara	1	Est	1.54124
Kara	2	Est	1.36369
Kara	3	Est	1.5781
AdviKara	1	Est	1.48501
AdviKara	2	Est	1.14411
AdviKara	3	Est	1.23182
Vyda	1	Est	1.21327
Vyda	2	Est	1.13855
Vyda	3	Est	1.25637
AdviVyda	1	Est	1.23052
AdviVyda	2	Est	1.5125
AdviVyda	3	Est	1.2898
Doma	1	Est	0.99827
Doma	2	Est	0.89247
Doma	3	Est	0.96887
AdviDoma	1	Est	1.22006
AdviDoma	2	Est	1.17177
AdviDoma	3	Est	1.15879
Roun	1	Est	0.88185
Roun	2	Est	1.1243
Roun	3	Est	1.03206
AdviRoun	1	Est	1.55158
AdviRoun	2	Est	1.35742
AdviRoun	3	Est	1.5683
Tran	1	Est	1.2274
Tran	2	Est	1.1214
Tran	3	Est	1.0307
AdviTran	1	Est	1.18059
AdviTran	2	Est	0.93407
AdviTran	3	Est	1.3262
Mix7	1	Est	0.6956
Mix7	2	Est	0.72411
Mix7	3	Est	0.69994
CK	1	AChE	1.07836
CK	2	AChE	0.93942
CK	3	AChE	0.98222
Advi	1	AChE	1.0061
Advi	2	AChE	1.0891
Advi	3	AChE	1.1356
Brac	1	AChE	0.9514
Brac	2	AChE	1.04091
Brac	3	AChE	1.03233
AdviBrac	1	AChE	1.02388
AdviBrac	2	AChE	0.89638
AdviBrac	3	AChE	0.86453
Kara	1	AChE	1.09028
Kara	2	AChE	1.10827
Kara	3	AChE	1.10751
AdviKara	1	AChE	1.06032
AdviKara	2	AChE	1.12009
AdviKara	3	AChE	1.11222
Vyda	1	AChE	1.12245
Vyda	2	AChE	1.18165

```
P450 Activity Data
input treat $ rep P450 @;
cards;
Control      1  0.98253
Control      2  1.01377
Control      3   1.0037
Advise43     1  1.15185
Advise43     2  1.26755
Advise43     3  0.99006
Advise22     1  1.54219
Advise22     2  1.09864
Advise22     3  1.02538
Advise56     1  1.41536
Advise56     2  1.44126
Advise56     3  1.58643
run;
```

```
Vyda         3 AChE    1.19632
AdviVyda     1 AChE    1.24013
AdviVyda     2 AChE    1.18747
AdviVyda     3 AChE    1.04973
Doma         1 AChE    0.76394
Doma         2 AChE    0.91727
Doma         3 AChE    1.03709
AdviDoma     1 AChE    1.07408
AdviDoma     2 AChE      0.932
AdviDoma     3 AChE      1.162
Roun         1 AChE    0.65413
Roun         2 AChE    0.82072
Roun         3 AChE    0.96413
AdviRoun     1 AChE    1.04058
AdviRoun     2 AChE    0.95515
AdviRoun     3 AChE    0.91132
Tran         1 AChE    1.03678
Tran         2 AChE    1.24818
Tran         3 AChE    0.98458
AdviTran     1 AChE    0.88231
AdviTran     2 AChE    0.89976
AdviTran     3 AChE     0.8868
Mix7         1 AChE    0.90944
Mix7         2 AChE    0.84642
Mix7         3 AChE     0.9802
CK           1 inverta 1.05756
CK           2 inverta 1.04855
CK           3 inverta 0.89389
Advi         1 inverta 1.25066
Advi         2 inverta 1.24839
Advi         3 inverta 1.31473
Brac         1 inverta 1.11543
Brac         2 inverta 1.03154
Brac         3 inverta 1.34472
AdviBrac     1 inverta 0.73199
AdviBrac     2 inverta 0.95224
AdviBrac     3 inverta  0.7181
Kara         1 inverta  1.2159
Kara         2 inverta  1.7801
Kara         3 inverta 1.06108
AdviKara     1 inverta 0.65723
AdviKara     2 inverta  1.0326
AdviKara     3 inverta 0.91335
Vyda         1 inverta 1.19016
Vyda         2 inverta 1.26871
Vyda         3 inverta 1.20359
AdviVyda     1 inverta 1.21482
AdviVyda     2 inverta 1.14216
AdviVyda     3 inverta 1.00382
Doma         1 inverta 1.01387
Doma         2 inverta 1.20497
Doma         3 inverta 1.44435
AdviDoma     1 inverta 1.54547
AdviDoma     2 inverta 1.19492
AdviDoma     3 inverta 1.02575
Roun         1 inverta 1.15483
Roun         2 inverta 1.04709
Roun         3 inverta 1.23095
AdviRoun     1 inverta 1.18993
AdviRoun     2 inverta 1.66891
AdviRoun     3 inverta  0.8872
Tran         1 inverta 1.06462
Tran         2 inverta 1.34105
Tran         3 inverta 1.65737
AdviTran     1 inverta 0.99044
AdviTran     2 inverta 1.13587
AdviTran     3 inverta 1.21034
Mix7         1 inverta 1.03396
Mix7         2 inverta 1.13533
Mix7         3 inverta 1.09474
run;
data a1; set a;
```
